# Compliance with and acceptability of two fortified balanced energy protein supplements among pregnant women in rural Nepal

**DOI:** 10.1111/mcn.13306

**Published:** 2021-12-15

**Authors:** Tsering P. Lama, Katie Moore, Sheila Isanaka, Leslie Jones, Juliet Bedford, Saskia de Pee, Joanne Katz, Subarna K. Khatry, Steven C. LeClerq, James M. Tielsch

**Affiliations:** ^1^ Nepal Nutrition Intervention Project—Sarlahi (NNIPS) Kathmandu Nepal; ^2^ Anthrologica Oxford UK; ^3^ Departments of Nutrition and Global Health and Population Harvard T.H. Chan School of Public Health Boston Massachusetts USA; ^4^ Department of Global Health Milken Institute School of Public Health George Washington University Washington District of Columbia USA; ^5^ Department of International Health Johns Hopkins Bloomberg School of Public Health Baltimore Maryland USA

**Keywords:** acceptability, balanced energy protein supplement, compliance, Nepal, pregnant women

## Abstract

Some evidence suggests that balanced energy protein (BEP) supplements taken during pregnancy and lactation can have positive effects on birth outcomes such as small‐for‐gestational age and birthweight, but more evidence is needed on the long‐term use and acceptability of such supplements. We conducted a mixed‐methods formative research study to assess and compare compliance with and acceptability of two BEP supplements, a lipid‐based peanut paste and a biscuit, to identify BEP supplements for subsequent inclusion in an efficacy trial. We conducted an 8‐week feeding trial of daily supplementation among two groups of 40 pregnant women each in rural Nepal. Compliance data were collected and supplements distributed at the weekly visits. Sensory properties of the supplements were assessed using a 7‐point Likert scale. In addition, in‐depth interviews with women (*n* = 16), family members (*n* = 6) and health workers (*n* = 6) and focus group discussions (FGDs) (*n* = 4) were conducted to explore themes related to general use and intention of future use of the supplement. Overall self‐reported compliance was high: medians of 91.1% in the lipid‐based peanut paste group and 96.4% in the biscuit group. Both supplements were rated highly on overall likability (median score 6/7) and sensory properties. Qualitative findings showed that sustained use of the supplements was attributed to expected health benefits, favourable sensory attributes, and family support. The FGDs suggested providing the option to choose between more than one type/flavour of supplements to improve compliance. Sharing was mostly evident in the first week with higher sharing reported in the biscuit group.

## Key messages

- Both the lipid‐based peanut paste and the vanilla biscuit were well accepted by pregnant women in rural Nepal during an 8‐week home feeding trial.
- The compliance measurements showed high overall compliance in both supplement groups with the reasons being the perceived health benefits to the mother and baby, favourable sensory properties, enabling environment at home for product consumption (privacy and family support) and ease of product use.
- Before an efficacy trial of a nutritional food supplement, it is important to conduct a mixed‐methods feeding trial in the study population to understand the contextual factors that enable and prevent the use of supplements to inform ways to improve compliance with the supplements.

## 1 INTRODUCTION

Low birthweight (LBW, <2500 g in a livebirth) is associated with higher risks of child mortality, stunting, developmental delays, and adult‐onset metabolic diseases (Christian et al., 2013; Gu et al., 2017; Jornayvaz et al., 2016; Victora et al., 2008). The two main underlying causes of LBW are small‐for‐gestational age (SGA) and/or preterm birth (gestational age < 37 weeks) (World Health Organization, 2014). Annually, 23.3 million babies are estimated to be SGA (A. C. C. Lee et al., 2017), and 14.8 million are preterm (Chawanpaiboon et al., 2019). In 2015, the prevalence of LBW in South Asia was 26.4% and made up almost half of the 20.5 million babies born LBW globally (Blencowe et al., 2019).

The World Health Assembly's proposed target of a 30% reduction in LBW by 2025 (World Health Organization, 2014) will not be met unless progress in maternal nutrition and health is accelerated (Blencowe et al., 2019). Deficiencies in protein, energy and micronutrients during pregnancy are linked to restricted foetal growth resulting in adverse birth outcomes such as LBW and SGA (Abu‐Saad & Fraser, 2010; Da Silva Lopes et al., 2017; Fall et al., 2009; Gernand et al., 2016). One of the interventions recommended by WHO to improve maternal nutritional status is antenatal balanced energy protein (BEP) supplementation in settings where the prevalence of maternal low body mass index (BMI, <18·5 kg/m^2^) is 20% or more (World Health Organization, 2016). This is based on findings from systematic reviews of BEP supplementation during pregnancy that demonstrate moderate levels of evidence that such dietary supplementation can decrease the risk of stillbirth and SGA births and increase birthweight (Imdad & Bhutta, 2011; Lassi et al., 2020; Ota et al., 2015; Stevens et al., 2015). However, heterogeneity across studies such as in the form of BEP supplements (energy content, nutritional composition), the control intervention, the study population and outcome definition, and moderate to low quality of evidence call for careful interpretation of the findings (Bill & Melinda Gates Foundation, 2017; Imdad & Bhutta, 2011; Ota et al., 2015; Stevens et al., 2015). In addition, more evidence of the effect of BEP supplements combined with multiple micronutrients and the effect on long‐term growth of a child is needed. Thus, in 2016, the Bill & Melinda Gates Foundation (BMGF) convened an expert group to recommend the optimal nutritional composition of a BEP supplement. The group also recommended research on the acceptability and use of fortified BEP supplements by pregnant and lactating women (PLW) and proposed trials assessing their impact on child health outcomes in low income contexts (Bill & Melinda Gates Foundation, 2017).

A large randomized control trial was designed to test the efficacy of fortified BEP supplements with nutrient content consistent with the BMGF guidance during pregnancy and lactation on pregnancy and child health outcomes in rural Nepal (ClinicalTrials.gov Identifier: NCT03668977). This is similar to a sister trial in Burkina Faso Micronutriments pour la SAnté de la Mère et de l'Enfant (MISAME) III study (ClinicalTrials Identifier: NCT03533712). The MISAME III was also designed as a three‐phase study with the first phase (Jones et al., 2021) and second phase (de Kok et al., 2021) being formative research to inform the design of the larger efficacy trial (Vanslambrouck et al., 2021).

Before the efficacy trial, a two‐phase formative study was designed to assess the acceptability and utilization of BEP supplements in the same population, to determine which supplements might best promote optimal compliance in the efficacy trial. In the first phase of the formative study, we conducted a single‐meal test to describe the general preferences/acceptability across 11 different BEP supplement types and flavours (Lama et al., 2021). Presented here is the second phase of the formative study which aimed to assess the compliance with and acceptability of the two short‐listed supplements from the first phase through a mixed‐method 8‐week home feeding trial of daily supplementation among pregnant women. The BEP supplement found to be most acceptable and utilized from this formative research would then be used in the larger efficacy trial in the same study population.

## 2 METHODOLOGY

### 2.1 BEP supplements

This study was the second part of a three‐phase research project to compare the incidence of SGA newborns born to mothers randomized to receive either a daily BEP nutritional supplement with multiple micronutrients or control during pregnancy in Nepal. The two highest ranking supplements identified in the first phase were evaluated in this second phase of the formative research for midterm 8‐week acceptability and at‐home consumption. For this second phase study, two BEP supplements manufactured by Nutriset S.A.S. in France were used: a lipid‐based peanut paste in a single sachet serving of 72 g (389 kcal; 14.5‐g protein) and a vanilla biscuit, served in a package of six biscuits, with a total portion size of 75 g (375 kcal; 16.5‐g protein).

### 2.2 Study area and participants

This study was conducted in rural Sarlahi district, located in the southern plains of Nepal. Bordering India, Sarlahi district has a population of 769,729 and is located in Province 2 of Nepal (Government of Nepal & Central Bureau of Statistics, 2012). Among the seven provinces of Nepal, women aged 15–49 in Province 2 had the highest prevalence of underweight (BMI < 18.5 kg/m^2^) at 29% and anaemia at 58% (Ministry of Health and Population [MOHP], 2017. Province 2 also had the highest prevalence of wasting, underweight and stunting among children under 5 years of age at 14.4%, 36.8% and 37.0%, respectively (Ministry of Health, Nepal et al., 2017).

The study population in Sarlahi district consisted of an undernourished pregnant women population. A previous large trial in this population, conducted in 2012–2017 (ClinicalTrials.gov, NCT01177111) found that among >28,000 enroled pregnancies, 37% were underweight based on early pregnancy BMI measurements, and the incidence of LBW and SGA was 29.4% and 46.8%, respectively (data not published). The typical daily diet in Sarlahi like in most parts of Nepal consists of rice (main staple), lentils and some seasonal vegetable dish with a pickle dish consumed twice daily by most people. Previous research in this study population showed that rice intake can be reduced during pregnancy; which could be attributed to an aversion to food, lack of appetite while for most protein and micronutrient‐rich foods, the majority reported eating the same quantities during pregnancy (Christian et al., 2006). Another study examined the micronutrient status of women in early pregnancy in this study area and found deficiencies in Vitamins A, E, D, riboflavin, B‐6, B‐12, folate, zinc, iron and copper. More than 80% of the women in the study were deficient in two or more micronutrients; the authors suggested that these deficiencies likely reflected dietary inadequacy before pregnancy (Jiang et al., 2005).

This study was conducted from May to August 2019 in two municipalities in Sarlahi district: Ishwarpur and Chandranagar. Forty pregnant women from each municipality, totalling 80 women, were purposively sampled and enroled in this study. The inclusion criteria for women recruited to the study were: married; age (15–40 years); gestational age (13–28 weeks); and residing in the study area for at least two months. The efficacy trial will supplement pregnant women starting from their second trimester at 14 weeks as we don't want early miscarriages to be blamed on the supplement. We therefore restricted enrolment in this pilot trial to similarly enrol women from 13 weeks but who would not deliver before the end of the 8‐week pilot follow‐up. Women with known allergies to one or more of the product ingredients (soy, dairy products, eggs, gluten and nuts) were excluded from participation. In addition to the pregnant women, a small number of other stakeholders such as family members of the pregnant women and health workers were also included in this study to gain their opinion and perspectives on the BEP supplements. The implementation of the data collection and field work was carried out by the Nepal Nutrition Intervention Project—Sarlahi (NNIPS), which is a long‐running research effort headed by investigators in the Department of International Health at Johns Hopkins Bloomberg School of Public Health in collaboration with Nepal Netra Jyoti Sangh, a national‐level Nepali nongovernmental organization, under the auspices of the Social Welfare Council of the Ministry of Women, Children and the Elderly of Nepal.

### 2.3 Study design and procedure

Over the course of the 8‐week period, the women were divided into two supplement groups (lipid‐based peanut paste and vanilla biscuit). The groups were assigned based on their geographical location (i.e., municipality) to avoid bias related to social interaction and for ease of supplement distribution. Participants from Ishwarpur municipality were assigned the vanilla biscuit and participants from Chandranagar municipality were assigned the lipid‐based peanut paste. A recruitment list of women meeting the inclusion criteria was created with names, ages, and gestational ages collected by female field team members based in the community in those two municipalities. From the larger recruitment list, a purposive sample of 40 pregnant women from each municipality (total 80 pregnant women) was selected based on their reported age, gestational age and geographical location (wards within the municipality). Trained NNIPS staff visited the women who met the inclusion criteria and provided details of the study. They also informed them about the risks, benefits and use of supplements. Informed consent with signature or thumbprint was then obtained, and a socio‐demographic and pregnancy history questionnaire was administered. Upon enrolment, all participants were given instructions on how to eat the supplements they were assigned, such as to eat one packet/serving each day in addition to their meals/snacks, to not share with others, and to keep both the used and the unopened packets of BEP supplements with them for subsequent collection. The NNIPS staff then provided a 1‐week supply of assigned supplements (i.e., eight supplement packets). Throughout the trial, during the weekly visits one extra packet was provided to ensure the daily dose is not missed due to loss or damage to the supplement, or a missed visit on the scheduled day. Only one extra supplement was provided every week and extra remaining packets (uneaten packets) from the previous week were collected.

Eight trained NNIPS staff, all local village women (four from each municipality) were assigned ten pregnant women each, and they visited the homes of the assigned women every week to deliver the supplements and document compliance to the recommended daily consumption of the supplements using a structured questionnaire‐based interview. The questionnaire asked about supplement consumption for the past 7 days (portion size consumed, timing, meal replacement and sharing), recorded the number of unopened packets from the past week and recorded the number of products provided for that week. If the supplement was not consumed, the reason was also obtained. In each weekly visit, the village woman staff person would ask the pregnant woman if she planned to be away from home the following week and if so, a 2‐week supply of supplements was given ahead of time. In the event the woman was not met in person for the weekly visit, consumption information was also obtained via phone interview when possible.

At the end of week 8, the women were asked to rate the supplement on its hedonic properties (colour, taste, texture, smell and overall appreciation) using a 7‐point Likert scale (1 = *Dislike it very much* to 7 = *Like it very much*). The women also rated their perception of product use in terms of being convenient to eat (1 = *Very difficult* to 7 = *Very easy*), product being medicine or food or both, and feeling full after eating a full serving. In addition, data were collected on willingness to consume these supplements every day for up to a total of 12 months (i.e., 6 months of pregnancy starting from the second trimester to 6 months post‐partum) using a 5‐point Likert scale (1 =* Definitely would not eat every day* to 5 = *Definitely would eat every day*). We also explored perceptions and acceptability of alternate products amongst pregnant women. The women were given an opportunity to taste three additional flavours of the supplement type assigned to them: lipid‐based paste was in cocoa, vanilla and strawberry flavours and the biscuit flavours were strawberry, orange and almond. Participants quantified their appreciation of each new flavour's colour, taste, and smell as well as their overall appreciation for each flavour, using a 7‐point Likert scale that ranged from 1 (*Dislike very much*) to 7 (*Like very much*).

To get contextual information on the diets of pregnant women in general, their experience with using the supplements and their future use of the product to which they were assigned, a sub‐sample of eight women in each supplement group were asked to participate in one‐to‐one in person in‐depth interviews (IDI) after 4 weeks of product use. The women were purposively selected to include various age groups (<20 years and ≥20 years) and gestational ages.

Furthermore, another sub‐sample of the enroled women who were not part of the IDIs were asked to participate in focus group discussions (FGDs) soon after the end of 8‐week trial. The FGDs sought to understand the women's experiences with the BEP supplements, factors that influenced acceptability and compliance, potential future use of supplements during pregnancy and opinion on additional flavour profiles or alternate supplements. Two FGDs for each supplement group (5–8 women each) were conducted in a private setting at a central location in the study area. The IDIs and FGDs were conducted by NNIPS female study team members trained in conducting qualitative interviews and FGDs using semi‐ structured interview guides (see Supporting Information File 1).

In addition, stakeholders such as family members (*n* = 6, 3 per group) and health professionals (*n* = 6) were identified and interviewed using specific semi‐structured interview guides to understand their attitudes towards supplementation during pregnancy.

All data collection activities were conducted in the local language, Maithili. Data for the quantitative forms were recorded or entered using the Research Electronic Data Capture (REDCap, © Vanderbilt University) application on a password‐encrypted mobile android device. The IDIs and FGDs were audio recorded, transcribed verbatim into Nepali using the notes and audio recordings by the interviewer, and then translated into English for analysis.

### 2.4 Data analysis

Stata version 15.0 (StataCorp.) was used for the quantitative data analysis. Participant characteristics, including socio‐demographic and pregnancy characteristics, were summarized using medians and interquartile ranges (IQR) for continuous measures and using counts and proportions for discrete measures. The 7‐point hedonic scale responses rating sensory properties, and responses to the Likert‐scale responses for the themes ‘acceptability’, ‘perception of product use’ and ‘willingness to use for 12 months’ were presented as medians (IQR).

Compliance was calculated in three ways: (i) packet count method, (ii) women's self‐reported consumption of full packet (serving) and (iii) women's self‐reported consumption of any amount (partial or full serving) (see Supporting Information File 2). For the packet count method, the total number of packets used was divided by the follow up time; this analysis was restricted to only those women who were met in person every week, as a record on packet count was not available for those not met in person. For the self‐report method, we calculated the overall compliance in terms of women's self‐reported consumption of a full serving and self‐reported consumption of any portion of a day's serving over the follow‐up period. The mean and median with IQR of the three different compliance measurements were compared between the two supplement groups. In addition to the overall compliance, weekly compliance rates were also calculated for the three measurements in both groups and compared with assess for any time trends.

A full analysis of the qualitative data was conducted using an inductive, thematic approach developed specifically for data generated through applied qualitative research. Dominant themes were identified through the systematic review of material and salient concepts were then coded by hand and/or using the Dedoose software programme and cross‐referenced by the research team for quality assurance. The emerging trends were critically analysed according to the research objectives to assess the 8‐week acceptability and at‐home consumption of the two selected BEP supplements. The findings from the qualitative and quantitative data were triangulated which further validated the results.

### 2.5 Ethical approval

Written informed consent was obtained from all the respondents. In addition, signed informed consent from a guardian or husband was obtained for participants under 18 years of age as per the research ethics requirement of the local ethics review body. Ethical approval was obtained from The Johns Hopkins Bloomberg School of Public Health Institutional Review Board (Baltimore, USA), George Washington University (Washington D.C., USA) and the Nepal Health Research Council, Ministry of Health and Population (Kathmandu, Nepal).

## 3 RESULTS

Background characteristics of the 80 pregnant women who participated in this study are presented in Table 1. As the two groups were not randomly assigned but assigned based on geographical location by municipalities, the distribution of educational background and religion varied. The lipid‐based peanut paste group located in Chandranagar municipality comprised of a mix of Muslim (42.5%) and Hindu (57.5%) religious backgrounds compared with the 100% Hindu background in the vanilla biscuit group (Ishwarpur municipality). A little over half (52.5%) the women in the lipid‐based peanut paste group reported never having attended school compared with 27.5% in the biscuit group. Other background and pregnancy characteristics were similar in the two groups (Table 1). By the end of the eight‐week home tasting trial, two women in the lipid‐based peanut paste group were lost to follow‐up (in‐person and via phone) as they had permanently moved out of the study district to India. During the 8‐week period, both groups had participants who were not met in person due to the participant being away that week and the interviewers collected compliance data through phone interviews for 16 out of 36 not met in person visits. In addition, six health professionals (three auxiliary nurse midwives, one health assistant, one pharmacist and one informal healthcare provider/local doctor) and six family members (two husbands, two mothers‐in‐law and two sisters‐in‐law) were interviewed.

**Table 1:** Socio‐demographic and pregnancy characteristics of study participants, by supplement group

	All	Lipid‐based peanut paste	Biscuit
*N*	80	40	40
Age, median (Q1–Q3)	22 (20.5–25)	22 (20–25)	22 (21.5–25)
Marital status, *n* (%)			
Married	80 (100)	40 (100)	40 (100)
School Attendance, *n* (%)			
None	32 (40)	21 (52.5)	11 (27.5)
Primary (1–5)	11 (13.8)	6 (15)	5 (12.5)
Secondary (6–10)	24 (30)	8 (20)	16 (40)
Higher secondary	13 (16.2)	5 (12.5)	8 (20)
Religion, *n* (%)			
Hindu	63 (78.8)	23 (57.5)	40 (100)
Muslim	17 (21.2)	17 (42.5)	0
Ethnicity, *n* (%)			
Madeshi	80 (100)	40 (100)	40 (100)
Household member, *n* (%)			
Mother/father	14 (17.5)	8 (20)	6 (15)
Brothers/sisters	12 (15)	6 (15)	6 (15)
Mother/father‐in‐law	56 (70)	28 (70)	28 (70)
Husband	76 (95)	37 (92.5)	39 (97.5)
Children	55 (68.8)	27 (67.5)	28 (70)
Brother/sister‐in‐law	55 (68.8)	28 (70)	27 (67.5)
Nephew/nieces	18 (22.5)	9 (22.5)	9 (22.5)
Other (grandparents)	5 (6.2)	3 (7.5)	2 (5)
Pregnancy history			
First pregnancy, *n* (%)	21 (26.2)	10 (25)	11 (27.5)
Number of living children, median (Q1–Q3)	1 (1–2)	1 (1–2)	1 (1–2)
Gestational age in months, median (Q1–Q3)	6 (5–6.5)	6 (5–7)	6 (5–6)
Number of antenatal consultations, median (Q1–Q3)	1 (1–2.5)	1 (0–2)	2 (1–3)

### 3.1 Supplement preference and hedonic evaluation

Table 2 shows the results of the hedonic scale responses rating the sensory characteristics (colour, taste, smell, texture) and overall appreciation of the two supplements. The results present consistently high appreciation for lipid‐based peanut paste and the vanilla biscuits on all metrics, with an overall appreciation median (IQR) score of 6 (6–7) for both products.

**Table 2:** Acceptability, perception and willing to use supplements after an 8‐week home trial period, by supplement group

	Lipid‐based peanut paste (*N* = 38)^a^, ^b^	Biscuit (*N* = 40)^b^
Appreciation of product (1 = *Dislike very much* to 7 = *Like very much*), median (Q1–Q3)		
Colour	7 (6–7)	6 (6–7)
Taste	7(6–7)	6 (6–7)
Texture	6 (6–7)	6 (6–7)
Smell	6 (6–7)	6 (5‐7)
Overall appreciation	6 (6–7)	6 (6–7)
Perceived child likeability	6 (6–7)	6 (6–7)
Perceived adult likeability	6 (5–7)	6 (4.5–7)
Perception of product use (1 = *Very difficult* to 7 = *Very easy*), median (Q1–Q3)		
Product is convenient to eat	6 (6–7)	6 (6–7)
Product is convenient to eat between meals	6 (6–7)	6 (6–7)
Product is medicine or food or both, *n* (%)		
Medicine	7 (18.4)	14 (35)
Food	28 (73.7)	20 (50)
Both a medicine and food	3 (7.9)	6 (15)
Feel full after full portion, *n* (%)		
Very full	16 (42.1)	17 (42.5)
Moderately full	18 (47.4)	18 (45)
Slightly full	4 (10.5)	5 (12.5)
Would use daily if provided, *n* (%)		
Definitely would eat every day	25 (65.8)	25 (62.5)
Probably would eat every day	9 (23.7)	9 (22.5)
Not sure if I would eat every day	3 (7.9)	3 (7.5)
Probably would not eat every day	0 (0)	2 (5)
Definitely would not eat every day	1 (2.6)	1 (2.5)

^a^ Two participants could not be met (in person or over the phone) at the end of the 8 weeks for the Hedonic Testing Form Interview as they had gone to India.

^b^ Two participants were interviewed over the phone at the end of 8 weeks as they had travelled outside the district.

In the qualitative interviews, although women in both groups responded positively to the taste overall, a few commented that the product was ‘very sweet’ or ‘too sweet’ and suggested it would be better if it had a little salt. Women in both groups proposed adding additional ingredients, such as salt or spice, or reducing the sugar content to improve taste. Identity (ID) 22 in the lipid‐based peanut paste group reported, ‘cashew, raisins, ground nuts, grams, ghee if all these would be there then it would be even better’. Many of the lipid‐based peanut paste group compared the taste to familiar ingredients such as nuts (cashews and almonds), chickpeas and raisins. A few women in the biscuit group noted a very strong aversion to the smell of the biscuits, describing it as a ‘stink’ and a smell like medicine: ‘Its taste is good, but it stinks. It smells like medicine… and it smells like pesticides which are used in green leafy vegetables’ (ID 71, biscuit group).

Women in both supplement groups reported that the supplements were easy to eat overall and in between meals (Table 2). The vast majority in both groups reported feeling moderately to very full after eating a full portion of the supplements. Similarly, a majority of women in both groups reported they would definitely or probably eat the supplements daily for up to 12 months if this product were recommended to them as beneficial for them and their baby and was provided for free. Only three women (7.5%) reported they would probably or definitely not eat the vanilla biscuit daily, and one woman (2.6%) would definitely not eat the lipid‐based peanut paste daily. These data were confirmed in one of the lipid‐based peanut paste FGDs where one woman said she might not eat it every day; another said that she would not be able to eat it at all because she did not like it.

A higher proportion of women in the biscuit group (35%) perceived the supplement to be medicine only compared with 18.4% in the lipid‐based peanut paste group, but the majority in both groups perceived the supplements as food (Table 2). Two pregnant women in FGD 2A biscuit group mentioned not liking the biscuit after eating it for a while as it smelled like medicine. Participant 2, in FGD 2A stated ‘Some medicines have some chemical smell, like that, I felt like that in the beginning. One feels like that having had the product in the beginning up to now’.

### 3.2 Compliance

Table 3 shows the overall compliance rates for both products for the three compliance measurements. For the packet count method, which was restricted to those who were met in person every week (*n* = 60/80), the mean overall compliance in both groups was high at 95.5% for lipid‐based peanut paste and 93.9% for the vanilla biscuit. The median and IQR of the compliance measurement for both groups were similar. The mean self‐reported consumption of a full portion and any portion of the required servings in both groups were similar at 88% and 93%, respectively. The median compliance for the biscuit groups was slightly higher using the self‐reported measurement (Table 3).

**Table 3:** Overall compliance over an 8‐week period, by supplement group

	Lipid‐based peanut paste	Vanilla biscuit
**Overall compliance over 8 weeks using various definitions:**	**Median (Q1–Q3)**, ***mean***	**Median (Q1–Q3)**, ***mean***
*Packet count method*	*N* = 26	*N* = 34
a. Overall compliance (%) over 8 weeks among those met in person	100 (94.8–100), *95.5*	100 (96.4–100), *93.9*
*Self‐reported method (non‐packet count method)*	*N* = 30	*N* = 37
b. Overall compliance of full portion only over 8 weeks among those met in person or over the phone	91.1 (85.7–98.2), *87.6*	96.4 (87.5–98.2), *88.4*
c. Overall compliance of any portion only over 8 weeks among those met in person or over the phone	98.2 (87.5–100), *92.9*	100 (94.6–100), *93.0*

**Figure 1:**
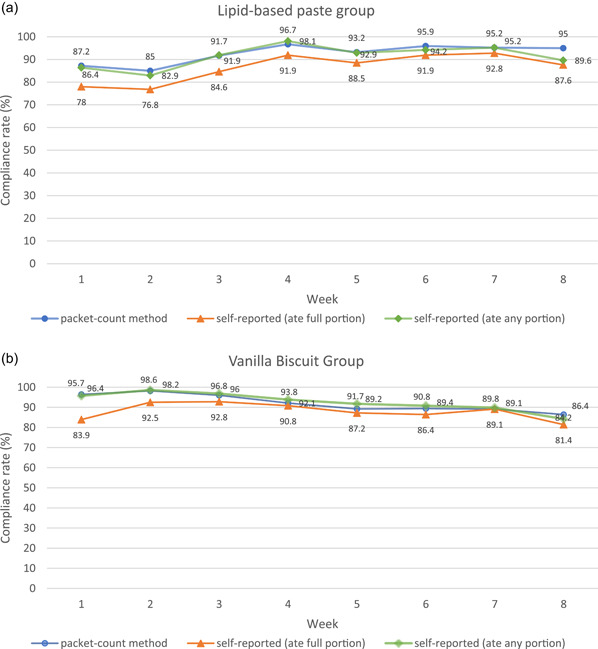
(a) Weekly median compliance rate during the 8 weeks for the lipid‐based peanut paste group. (b) Weekly median compliance rate during the 8 weeks for the vanilla biscuit group

According to the weekly compliance rates for the lipid‐based peanut paste (Figure 1a), compliance was lower in the first 2 weeks and increased in the later weeks, with the highest compliance seen in Week 4. In the qualitative interviews, many women in the lipid‐based peanut paste group reported changes in their perceptions of the products over time, mostly admitting to not liking the product in the beginning but getting used to it over the course of the 8‐week trial period:

Initially I didn't like it because it was hard to digest. Nowadays I am used to it and I like it (lipid‐based group, ID 18).

On the other hand, the vanilla biscuit group started with higher compliance in the first 3 weeks and then compliance decreased slightly towards the end (Figure 1b). The smell of the biscuits was not liked by a few in the biscuit group:

The product I liked this for the first week. But later after for some time, I started to dislike it.

I did not like it the smell of the product after eating it for a while (FGD2A, Participant 2, Biscuit group).

Interviews with family members aligned with feedback from the pregnant women, and most agreed that the pregnant woman in their household who was consuming the supplement (wife/daughter/daughter‐in‐law) had grown more accustomed to the product over time.

The qualitative findings from the in‐depth interviews and FGDs supported the quantitative findings and suggested that most women ate each product every day. Women reported consuming at least part of the supplement daily and in only a small number of cases were days of intake missed completely. A small number of women in both product groups recalled being unable to finish the full portion of the product every day, because they did not like it and/or they found it to be too much. This was supported by findings from the quantitative data regarding reasons for not consuming the product every day, where 22.5% of the lipid‐based peanut paste and 17.5% of the vanilla biscuit group reported that they ‘did not like it’, while responses for ‘not well’ and ‘other’ were recorded as 25% and 47.5%, respectively for lipid‐based peanut paste and 12.5% and 27.5% for the vanilla biscuit (Table 4).

**Table 4:** Overall sharing behaviour and reasons for not eating the products all days during the 8 weeks by supplement group

	Lipid‐based peanut paste	Biscuit
	*N* = 40	*N* = 40
Mean number of days there was sharing	0.4	1.0
Percent of participants who ever shared, *n* (%)	11 (27.5)	15 (37.5)
Ever shared with whom, *n* (%)		
Any child	8 (20.0)	14 (35.0)
Husband	1 (2.5)	2 (5.0)
Mother/mother‐in‐law	2 (5.0)	2 (5.0)
Sister/sister‐in‐law	1 (2.5)	2 (5.0)
Other	0 (0)	1 (2.5)
Reason for not eating the product all days, *n* (%)	*N* = 40	*N* = 40
Did not like it	9 (22.5)	7 (17.5)
Fasting	1 (2.5)	0 (0.0)
Lost/damaged	1 (2.5)	0 (0.0)
Not well	10 (25.0)	5 (12.5)
Other	19 (47.5)	11(27.5)
Reason for not eating food (meal/snack) as normally do	*N* = 51	*N* = 117
Too full	25 (49.0)	65 (55.6)
Nausea/not well	5 (9.8)	9 (7.7)
Not hungry	21 (41.2)	41 (35.0)
Other	0	2 (1.7)

### 3.3 Supplement consumption behaviour

The supplements were most often consumed in the morning; 48.3% of the lipid‐based peanut pastes and 45.6% of the vanilla biscuits were consumed before 12 PM (Supporting Information File 3). Women generally reported eating the supplement with breakfast, as a snack after breakfast or between other meals. A large majority, 82.5% of women in lipid‐based peanut paste group and 65.1% in the vanilla biscuit group, agreed that during the 8‐week tasting period they ate meals and snacks as they ‘normally would’ (supporting information file 3). Supplements were consumed both in a single sitting and in multiple sittings throughout the day, depending on personal preference, hunger, and ability to tolerate the product at a specific time. Quantitative data indicated that the vanilla biscuits were more frequently consumed over multiple sittings than the lipid‐based peanut paste (20.2% vs. 7.9%). This could be attributed to the composition of a full portion of vanilla biscuits (in which 6 individual biscuits needed to be eaten) rather than the lipid‐based peanut paste (where one dose equalled the full packet). A few women in the first FGD in the biscuit group mentioned not eating the biscuits in one sitting. Participant 3 in FGD2A said ‘Sometimes I finished one packet in two times, at that time I ate three pieces at one time and remaining at later times’. Similarly, participant 1 in FGD2A said ‘I used to take a whole day to finish a packet, I used to take out one piece, eat it and again take out one, like that, I used to finish’.

### 3.4 Factors influencing acceptability and compliance

#### 3.4.1 Diet during pregnancy

Women, their family members, and health workers all recognized the importance of a healthy and balanced diet to protect the wellbeing of pregnant women and their unborn children. Women indicated that they should eat well to ensure that they have sufficient energy. Foods such as lentils, leafy greens, and fruits and vegetables were highlighted as being particularly important for a nutrient‐rich diet during pregnancy. Women consistently emphasized that frequency of eating fruits and vegetables was dependent on seasonality and availability. Health workers and some family members emphasized the importance of including meats, fish, eggs, milk and other protein‐rich foods, as well as foods containing iron, calcium and potassium, in a pregnant woman's diet.

A majority of women indicated that they eat less food during pregnancy than before. Participants were asked whether the reduced intake was intentional [the phenomenon that has been recognized as ‘eating down’ (Christian et al., 2006)]; however, reasons cited for the reduction appeared to be linked primarily to other factors including reduced appetite, feelings of fullness and increased episodes of nausea. As one woman affirmed, ‘how can I eat a big amount, even after a small amount I feel discomfort’. The quantitative data also supports this, showing the feeling of fullness being the main reason for pregnant women in both groups not eating food as they normally did (Table 4). A larger proportion of women in the lipid‐based peanut paste group (82.5%) reported eating their food (meal/snack) as they normally did during the 8‐week trial compared with 65.1% in the biscuit group.

#### 3.4.2 Supplement awareness and use in the community

Both health workers and family members were familiar with vitamin supplements for pregnant women. Family members were aware of iron and calcium supplements and said that their pregnant family members were currently taking one or both. Health workers were more specifically aware of iron‐folic acid supplements and discussed use of vitamin A for post‐partum women. Some health workers also were familiar with multivitamins or B complex supplements. (This is presumably a reference to ‘R.B. TONE’ syrup, a product containing folic acid, vitamin B12, iron and calcium and advertised as a ‘Tonic for Red Blood Corpuscles’.) Health workers stated that pregnant women are given a 1‐month/30‐day supply of government‐supplied iron‐folic acid supplements; this was said by most to be to encourage women to return regularly to the health post but also potentially to avoid misuse of the product.

Questions regarding familiarity with and use of food supplements did not always appear to be well understood, particularly by family members and pregnant women. Only those few women who had been prescribed a protein supplement by a health worker were aware of such supplements. Several health workers had previously recommended use of protein supplements to pregnant women. Others, however, do not generally recommend food supplements. One said that it is not ‘within their jurisdiction’ while another said ‘We cannot recommend ready‐made protein. We need to go for natural health’. Those who recommend protein powder to pregnant women give them information on how to take the product and why it is necessary. They stated that women do appear to understand the information provided, based on follow up information obtained. One health worker noted that some women think that supplementary foods lead to fat babies and therefore difficult deliveries, but she says that they understand after counselling that this is not the case.

#### 3.4.3 Perceived health benefits

In the qualitative interviews and FGDs, when the women were asked if they would continue eating the supplements for the remainder of their pregnancy if provided to them, most of them said they would. The common perceived benefits of the supplements by the pregnant women and their family members were the health benefits for the women and their baby or that it gave strength/energy.

Why would I not eat [supplement], of course I will eat if it benefits, only there should not be bad things if it is good then people will eat by buying it also…it will be good for my body and for my child (ID 47, biscuit group).

Because it will provide strength to me that is why…It will give strength at the time of delivery (ID 30, lipid‐based peanut paste group).

The sister‐in‐law of ID 81 (lipid‐based peanut paste group) also perceived one benefit of the supplement to be ‘to gain energy, to increase the blood level in the body and for betterment of the baby’.

#### 3.4.4 Support of family members

Guardians (i.e., head of household member), mothers‐in‐law and fathers‐in‐law were reported to make most of the family‐level decisions about food purchase and preparation. Support of family members was an enabling factor for women to adhere to the supplement and not feel the need to share with others.

When asked if this product was advised to be consumed for the remaining of the woman's pregnancy period, would the family members want the product be shared with other family members or would they expect the woman to share this product, one mother‐in‐law (ID 14) responded ‘No, they won't expect. Why will they expect? It has been given for pregnant women, they should eat it. Why share?’.

One focus group participant highlighted the importance of providing information to family members, particularly the ‘guardian’ of the family: ‘First of all, the guardian must know and only then will they let you eat, and they will say ‘it is beneficial, do eat’; if the guardian is not informed then how will they let you eat?’ (FGD 2B, Biscuit Group, Participant 3).

When the women were asked if they would continue eating the supplement even if they had to buy it themselves, one woman in the lipid‐based peanut paste group reported ‘Will eat only if the guardian will buy and bring it’. When asked, some family members said they were willing to buy and give to the pregnant woman if available in the market; others stated lack of money to be a barrier to being able to buy it.

Husband of ID 34: ‘Yes, if it is beneficial why not’.

Sister‐in‐law of ID 81: ‘Sometimes we don't have money to purchase.…we want to feed her by purchasing but it will be a little bit difficult in family/house. If it is given free of cost, then we can say, it is given only for her and only she can eat this’.

#### 3.4.5 Role of health workers

Health workers' comments appear to reflect an overall receptiveness to, and potential support for, the concept of food supplements for pregnant women in this community. Several of the health workers said they would describe the product as ‘a very good thing’ and said they would recommend it because it is good for pregnant women and their babies. Another said that the product is good but would be better if it were given to all pregnant women. A few of the health workers were concerned that the product was not coming from the government and stated that in the future these food supplements should be provided for free through the health system (government) just like iron and calcium tablets.

During the FGDs, when asked where would be a good place to get these food supplements in the future, many women pointed to the government health facilities:

Best place to be available is in a hospital (referring to local health post), if we get free of cost we will eat. Who will provide free of cost besides the hospital? (Participant 2, FGD 1A, lipid‐based peanut paste group).

However, some health workers also clearly indicated concerns about side effects and the need to provide more detailed information and sensitization for health workers. One said, ‘This product is a good one’ and that he would encourage use if he had more information about its composition.

#### 3.4.6 Sharing practices

At the onset of the study, participants had been advised that the supplement was to be taken by pregnant women only, and women appeared to adhere to the directions given. Women reported limited sharing of the product over the course of the 8‐week testing period, with the average number of days of sharing in the lipid‐based peanut paste and biscuit group of 0.4 and 1 day, respectively. The percentage of participants who ever shared the products was higher in the biscuit group at 37.5% compared with 27.5% in lipid‐based peanut paste group. In both groups, women were more likely to share with children than with other members of the family (Table 4).

The understanding that the supplement was beneficial to the health of pregnant women and the perception of the supplement as a medicine helped women justify their sole consumption of the product: In‐depth Interview with ID 46, biscuit group: ‘No, I didn't share, sister. If children asked with me, I would say this is medicine. So, they don't eat assuming that it is medicine. I don't share. [Children] they eat lentil and rice, so they won't need this much, but if a pregnant woman eats only rice and lentil it won't be beneficial to her. This product is beneficial to the pregnant women. The pregnant women eat more those things which will be beneficial to them, because of that I eat this’.

Others avoided sharing by eating in privacy: ‘When they [mother‐in‐law and sister‐in‐law] go out of the home, I will be alone whole day only then I eat’ (ID 18, lipid‐based peanut paste group).

Once they advise us not to share, we won't share. If there are children around, I don't feel good to eat in front of them because of that I eat this product when children are not around (ID 71, biscuit group).

Women also suggested that the decision to share would be theirs alone and said that family members would ‘scold’ them if they were found to be sharing with others:

I gave him [my son] some of one packet, after he started to cry because he wanted that and once he tried to steal and eat it. But my husband scolded me and told me not to share with children, he asked me to eat the product [alone] and I have not shared after that day (ID 49, biscuit group).

Any sharing was mostly seen in the first week of the study and confirmed in some of the IDIs where, for example, one of the women mentioned sharing the biscuits in the beginning but reported she did not share after that:

Yes, when I got it on the first day, I shared with everyone and they asked to taste the product and everybody told that, it smells like medicine and they didn't like it but my daughter liked it… right now, I haven't shared with anyone (ID 73, biscuit group).

At the beginning I didn't like this product and I gave little bit to the child, the child asked for it and being a small child, I gave little bit, but now days I am eating without sharing with him (ID 25, lipid‐based peanut paste group).

#### 3.4.7 Supplement choice

Among the new flavour variants of the assigned supplements, those in the lipid‐based peanut paste group favoured the vanilla flavour and those in the biscuit group favoured the orange flavour with 86% and 89.4% reporting they would definitely or probably eat the product every day. In contrast, the cocoa lipid‐based peanut paste and the almond biscuits had the highest reports of definitely would not eat every day at 13.9% and 13.1%, respectively.

Between the two supplements, almost all participants in the lipid‐based peanut paste FGD group preferred the biscuit over the lipid‐based peanut paste: ‘It is delicious’; ‘The taste is good; the smell is also good, and it is good to eat’. In the biscuit FGD groups, participants also preferred the biscuit over the lipid‐based peanut paste, and a few said they did not like the taste of the lipid‐based peanut paste:

If lipid‐based peanut paste was given to us, we might not have eaten the product (Participant 6, FGD 2A, Biscuit Group).

However, in another FGD, participants liked the lipid‐based peanut paste as it was new and found it similar to ‘Halwa’ a sweet pudding. Another said she might like the lipid‐based peanut paste after eating it for a while, as had been the case with the biscuit: ‘At first I had not even liked the biscuit but liked it after eating continuously, This [lipid‐based peanut paste] I have been just eating that's why I did not liked it, if I keep on eating then will like it’ (Participant 3, FGD 2B, biscuit group).

Focus group participants were asked a series of questions about being allowed to change products or flavours periodically if they were to use the supplements in the future. Most women wanted to have different flavours or products available, with the majority wanting to change weekly:

You like it [biscuit] now in the beginning but later will not like this same food product (Participant 6, FGD 1B, lipid‐based peanut paste).

Eating the same food, one does not get satisfied. Would like to eat different flavours (Participant 1, FGD 1B, lipid‐based peanut paste).

If the same biscuits were provided in different tastes, I would eat. I would eat until six months after delivery; if provided in the same flavour, then I would not be able to eat (Participant 6, FGD 2A, biscuit).

The option to change the flavours of supplements was also suggested by one of the husbands when asked if he would like his wife to continue eating the supplement: ‘Yes, I want but if the taste is different. If it is always the same taste, the person will not be satisfied. Sometimes, if we eat same taste again and again, we don't like to eat because of that' (Husband of ID 34, lipid‐based peanut paste group).

## 4 DISCUSSION

### 4.1 Comparison of our findings to other studies

Our results suggest that both BEP supplements were well accepted and had high overall levels of compliance with little variation between the supplements. The weekly compliance of the lipid‐based peanut paste group was slightly lower initially but increased over time while compliance of the biscuit group started higher in the first 2 weeks and decreased slightly over time. However, the overall compliance over the 8‐week period was very similar in both supplements similar to the Burkina Faso sister study site that compared the same supplements in a medium‐term home feeding trial (de Kok et al., 2021). The MISAME III phase two study had higher self‐reported full compliance at 99.8% and 99.6% for the lipid‐based peanut paste and the vanilla biscuit, respectively (de Kok et al., 2021). This could partly be due to the randomized cross‐over study design in which women received a continued weekly supply of each supplement for 4 weeks, which is shorter than the 8‐week duration in our study. Our medium‐term compliance results for both supplements were similar to the median self‐reported compliance of small quantity lipid‐based nutrient supplements (LNS) for pregnancy of 83.7% and 93.8% in Ghana and Malawi, respectively (Klevor et al., 2016). An increase in self‐reported compliance over time was seen in other long‐term adherence studies of supplements in pregnant women in Niger (Clermont et al., 2018) and Malawi (Klevor et al., 2016). Similar to our findings, the women reported getting used to the product as one factor for increased compliance (Clermont et al., 2018). This study found that a large majority of women would definitely or probably would eat either product daily in the future if they were provided, with very little distinction between the two products. Reasons for continued future use were the perceived health benefits for the mother and baby and that they liked the supplement. This was similar to other studies (Adu‐Afarwuah et al., 2011; de Kok et al., 2021; Janmohamed et al., 2016; Klevor et al., 2016; Mridha et al., 2012). Other earlier studies have reported a high level of utilization, particularly when supplements are provided free of charge (Seck & Jackson, 2008). Support from family members and healthcare professionals could also play a key role in compliance and uptake of supplements. In Niger, health workers played an important role in sensitizing women and their families, thus increasing the acceptance of the supplements (Clermont et al., 2018).

This study suggests that the sensory properties of both the supplements were broadly accepted. Among the sensory properties, a few women in the biscuit group disliked the smell of the product and associated it with the smell of medicine. A strong aversion to the smell of products was one of the primary reasons for not liking the supplement, similar to other supplementation studies among pregnant women in two studies in Bangladesh using peanut‐based ready‐to‐use therapeutic food (PlumpyNut®) (Ali et al., 2015) and LNS (Harding et al., 2014) and a small quantity‐LNS study among PLW in Ghana (Klevor et al., 2016). A study of a peanut‐based ready‐to‐use therapeutic food (PlumpyNut®) among malnourished PLW in Bangladesh revealed low acceptability, citing unfavourable taste (60%) and smell (43%) of the product, and called for re‐examining the use of peanuts as a core constituent in supplements in the South Asia context (Ali et al., 2015). In contrast, our findings highlighted the high acceptance of the taste and smell of the lipid‐based peanut paste.

The notion of ‘choice’ and the option of having a choice was identified as an important factor when considering future product utilization. This was consistent with existing research on prenatal supplement acceptability and adherence in the sister study in Burkina Faso (de Kok et al., 2021) and also in the formative first phase of the study in Nepal (Lama et al., 2021). There have been limited trials of BEP supplements where an option to choose between two or more supplements and/or flavour profiles has been provided and this could be an important consideration in future interventions (Lama et al., 2021).

Sharing of the supplement with household members was not frequently reported but was higher in the biscuit group. Women highlighted limited sharing of the product over the course of the 8‐week testing period and any sharing that was reported was primarily with women's children and in the earlier weeks, as in other settings (Clermont et al., 2018; Klevor et al., 2016). Strong household‐level support and appreciation for the instruction not to share was reported by women and family members with lower levels of sharing. The understanding that the supplement was beneficial to health and the perception of the supplement as a medicine justified not sharing the supplement as seen in another supplementation study in Burkina Faso (Iuel‐Brockdorf et al., 2016). Some women reported eating the supplement only when alone, similarly in Niger (Clermont et al., 2018). While all participants were instructed to eat the supplements in addition to their normal meal/snack, the biscuit group had a higher proportion of women reporting not eating their normal meal/snack. Our findings showed that feeling too full or not hungry were reasons for not eating food (meal/snack) as they normally do, which may indicate the use of the food supplement replacing the normal diet. Home observations or diet recall studies are needed in the efficacy trial to better understand these practices. Clear instructions on use of supplements and standard messages that discourage sharing of supplements and reducing normal food intake are important to increase compliance in future trials and programmes.

### 4.2 Strengths

Study strengths included the mixed‐methods design which provided confirmability in findings across qualitative and quantitative data. The combination of quantitative hedonic test questions with the qualitative methods allowed for a unique and in‐depth understanding and exploration of themes and the rich contextual information obtained triangulated quantitative results. In addition, there are significant gaps in the current literature on the family‐ and social‐level factors that influence women's supplement use and practices which this study helps fill. The length of the home feeding trial is another strength of our study, as it was longer than some other acceptability studies that were limited to 14 days which may not reflect long‐term acceptability (Adu‐Afarwuah et al., 2011; Isanaka et al., 2019; Mridha et al., 2012).

### 4.3 Limitations

Study limitations included the possible influence of social desirability bias, an inherent risk when conducting rapid data collection (Fisher, 1993). In this study, most measurements of acceptability, sharing and compliance were self‐reported. It is possible that participants may have expressed answers they perceived to be appropriate; this may have resulted in underreporting of negative responses to attributes of the BEP supplements as well as adherence and sharing practices. Participants were encouraged to speak openly and honestly, and the frank and sincere dialogue elicited from participant discussions suggested that such bias was minimized. Findings were also triangulated across participant groups and with quantitative data to test the validity of answers. In addition, we note that Likert scores on organoleptic properties varied only slightly between the products, with predominantly positive median scores in the 6 (‘Liked moderately’) to 7 (‘Liked very much’) range. This may reflect the limitations in using a Likert scale to assess supplement acceptability in a cultural context where people are unfamiliar with the Likert scales and may be unwilling to provide negative feedback (Flaskerud, 2012; J. W. Lee et al., 2002). Another limitation to our study was the design of single product use assigned to each group. As the women in the lipid‐based peanut paste group showed a preference for the biscuit during the FGDs, a cross‐over design like the one used in the Burkina Faso study (de Kok et al., 2021) would have provided more insight into the preference between the two supplements.

### 4.4 Implications for future work

The present research provided valuable insights to inform a future efficacy trial of these BEP supplements and considerations for future product development. Our study assessed medium‐term acceptability and compliance over 8 weeks, which does not reflect the long‐term compliance and acceptability of a single product for 6 months or more, as would occur in a larger efficacy trial of supplementation during pregnancy and lactation. Thus, the compliance and product acceptability measured in a larger efficacy trial will help assess behavioural fatigue that could arise from long‐term daily use of BEP supplements. Given the preference expressed for different products or flavours, providing a choice in a longer trial may reduce such fatigue. In the efficacy trial, we will be exploring potential displacement of health foods and replacement of meals/snacks through maternal diet questionnaire on all participants and a 24‐h diet recall in a subset of women in both groups (supplement and non‐supplement). In addition, clear detailed counselling on nutrition during pregnancy to all participants and counselling on the BEP supplement and its use will be provided to those in the supplement group in the efficacy trial. The importance of the larger efficacy trial is that appropriate food supplements could be especially valuable to the health of pregnant women in places where food insecurity exists and also where unhealthy processed foods are becoming more available. If the efficacy trial shows a health impact of supplementation, there will be additional work to turn this into a programme, including whether to provide the supplement free of charge or market through commercial channels, converting the product into a locally produced food, how to educate women about the value and use of the product and how to make sure such a product is not confused with unhealth snack foods that are increasingly being marketed in places like Nepal.

## 5 CONCLUSION

This study was the first to assess a medium‐term acceptability and compliance of BEP supplements among pregnant women in Nepal. Both the lipid‐based peanut paste and vanilla biscuit had high compliance and acceptance, with positive responses on willingness to use for longer duration if provided. The common drivers for compliance with and acceptability of the supplements were the perceived health benefits to the mother and baby, favourable sensory attributes, ease of use and support of family members. Achieving acceptance and continued consumption of either BEP supplement in the larger efficacy trial may require health promotion messages, clear instructions, regular monitoring and if possible, providing the option to choose between more than one product.

## CONFLICT OF INTERESTS

The authors declare that there are no conflict of interests.

## AUTHOR CONTRIBUTIONS

SP, SI, JB, LJ and KM conceived the study design and developed the study tools. All authors contributed towards the review and finalization to the study design and study tools. TPL, KM, LJ, SKK and SCL were involved with the training of the data collectors. TPL and SKK led the field team and supervision of the data collection. TPL analysed the quantitative data and prepared the first draft of the manuscript. KM and LJ analysed the qualitative data. SI, JMT, JK, SP, SCL and JB were involved in the interpretation of the data. TPL and KM drafted the manuscript. All authors were involved in the critical revisions of the manuscript for important intellectual content. All authors approved the final draft of the manuscript.

## Supporting information

Supporting information.

Supporting information.

Supporting information.

## Data Availability

The data is not shared in this manuscript but deidentified data can be made available upon request.
