# Supplementary material for: Compliance with and acceptability of two fortified balanced energy protein supplements among pregnant women in rural Nepal
Source: Matern Child Nutr. 2021 Dec 15;18(2):e13306. doi: 10.1111/mcn.13306 (PMC8932730; doi:10.1111/mcn.13306)
Supplement: Supplementary file 1 — Supporting information. [file MCN-18-e13306-s003.pdf]

Supporting information file 1: In-depth Interview and Focus Group Discussion Guides

<u>In-depth interview guide: pregnant women</u>

1 <u>Pregnancy and the pregnant woman's role in the household</u>

1.1 Has your duties / activities changed since becoming pregnant (compared to before pregnancy)? How?

2 <u>Diet during pregnancy</u>

2.1 What kinds of food do you think a nutritious diet during pregnancy should include?
- What fruits and vegetables do you eat? How often (how many times a day/week/month)? Does this differ by season?
- Do you eat any animal products such as meats, fish, milk, eggs, etc.? How often (how many times a day/week/month)?

2.2 What are the specific foods you believe you should eat during pregnancy? What are the specific foods you believe you should not eat during pregnancy?
- Do concepts of foods that make you feel 'hot' or foods that make you feel 'cold' influence what you eat during pregnancy? What foods?

2.3 Are there foods or drinks you have added to your diet since becoming pregnant? What? Why? (tradition/craving/other reasons)
- Are there foods or drinks you have removed from your diet since becoming pregnant? What? Why? (tradition/physical aversion/other reasons)

2.4 When you are pregnant, do you receive more food and drinks or less food and drinks than normal? If different from normal, in what way and why?

2.5 Do you try to restrict the amount of food you eat during pregnancy?
- If yes, why? If yes, how much do you restrict it? From whom/where did you hear about restricting food in pregnancy?

2.6 Who within your family decides what foods and how much you eat during pregnancy?

2.7 Do you eat snacks during your pregnancy?
- If yes, what do you eat? Where, when and how often do you eat snacks?
- Do you share your snacks with members of your family? Is it expected that you will share? How do you know?

3 <u>Availability and access to supplements</u>

<u>*(Note: Ask the participant to bring any pregnancy related medicines and food/nutrition supplements that she is taking or has been prescribed including empty containers/boxes/strips)*</u>

3.1 Before this study, did you know about iron or vitamin supplements for pregnant women?
- If yes, what type? Did you take them? Why / why not?

3.2 Before this study, were iron or vitamin supplements specifically for pregnant women available in your community? If yes, what type? When do women take them? Where do women get them?

3.3 Before this study, did you know about food supplements for pregnant women?

- If yes, what type? Did you take them? Why / why not?

3.4 Before this study, were food supplements specifically for pregnant women available in your community? If yes, what type? When do women take them? Where do women get them?

4 **<u>Product Preference (Show product that this woman was assigned; Plumpy Mum, or Biscuit)</u>**

4.1 Tell me about this product. What do you like? What do you like most about this product? Why?

4.2 What do you dislike? What do you like least about this product? Why?

4.3 Is this similar to other food / drink that you've eaten before or you are familiar with?

- If so, what food and why (flavour/texture/color/smell/ etc.)?
- Do you like that eating such 'xxx' food/drink during pregnancy?

4.4 What are your suggestions to make this product better? (Flavour/format/texture/convenience etc.)

4.5 Have you shared this product with your family members?

- If yes, who? What did they think?
- How much did you share? Has this increased or decreased since you started to eat the product?

4.6 Has your opinion of the product changed whilst you have been using it? If yes, how?

4.7 Have your family's opinions of the product changed whilst you have been using it? If yes, who / how?

5 **<u>Experience of use (during study)</u>**

5.1 While you have been eating this product, have you eaten it every day? If no, why not?

- Have you been eating the full serving every day? If no, why not?

5.2 How have you been eating this product? (as a snack, as part of a meal, between meals)

- While you have been eating this product, has it been
  a) replaced a whole meal/snack;
  b) replaced some of a meal/snack; or
  c) been an addition to the food you normally eat at a meal/snack (extra)?
- If it has replaced all or some of the food you normally eat, which food, when (i.e. what meal did it replace) and why?

5.3 While you have been eating this product, did you eat the entire serving at one time, or did you split it up into smaller portions to eat throughout the day? What time(s) of day did you eat the food? Why?

5.4 Is this food easy for you to eat in your home setting? Why/ why not?

5.5 How have you been feeling overall since eating this product

6 **<u>Future Utilization</u>**

6.1 If this supplement was recommended for you because you are pregnant, would you share it with other family members for the rest of your pregnancy?

- Why or why not?
- Would your share of food at mealtimes be reduced because you have this product? If yes, by whom?

6.2 If this product continued to be given for free would you keep eating it for the rest of your pregnancy? Why / why not?

- How frequently would you eat it? Ex. Once a day, several times a day, several days a week, several days a month etc.
- If you had to pay would you continue to use it for the rest of your pregnancy? Why / why not?

Thank you and close

<u>In-depth interview guide: family members (husbands/mother-in-law/father-in-law)</u>

1 <u>Pregnancy and the pregnant woman's role in the household</u>

1.1 When your [wife / daughter (in-law) etc.] is pregnant, do her duties / activities change? How?

2 <u>Diet and food utilisation practices and beliefs</u>

2.1 Tell me about food and meals in your household

- How many times a day does your family eat meals? When?
- Who makes the decisions about what foods are purchased/prepared within your household?
- Who eats first/last? When does your wife/daughter (in-law) etc. eat?
- Who decides which family members get which foods, and how much they receive?
- How is food shared during meal? Who receives most/least food?

3 <u>Diet during pregnancy</u>

3.1 What kinds of food do you think a nutritious diet during pregnancy should include?

3.2 What are the specific foods you believe pregnant women should eat during pregnancy? What are the specific foods you believe pregnant women should not eat during pregnancy?

- Do concepts of foods that make pregnant women feel 'hot' or foods that make them feel 'cold' influence what what they eat during pregnancy? What foods?

3.3 Do women try to restrict the amount of food they eat during pregnancy?

- If yes, why?
- If yes, how much do they restrict it?
- From whom hear/know about restricting food in pregnancy?

3.4 Do women eat snacks during pregnancy?

- If yes, what do they eat?

4 <u>Availability and access to supplements</u>

<u>(Note: Ask the participant to bring any pregnancy related medicines and food/nutrition supplements that the pregnant woman is taking or has been prescribed including empty containers/boxes/strips)</u>

4.1 Before this study, did you know about iron or vitamin supplements for pregnant women?
- If yes, what type?

4.2 Before this study, were iron or vitamin supplements for pregnant women available?
- If yes, what type? Where do women get them?

4.3 Before this study, did you know about food supplements for pregnant women?
- If yes, what type?

4.4 Before this study, were food supplements specifically for pregnant women available?
- If yes, what type? Where do women get them?

4.5 Before this study, had any woman in your family ever taken a food or nutrition/energy supplement during pregnancy?
- If yes, what type? Why did she take it?
- Where did she get it? What did you think about it? Do you think it was it helpful?
- (If not already mentioned) Did she take an iron supplement? If yes, why? Where did she get it? What did you think about it? Do you think it was it helpful?
- (If not already mentioned) Did she take a vitamin supplement? If yes, why? Where did she get it? What did you think about it? Do you think it was it helpful?
- If she has never taken a supplement (of any type) why not?

5 Experience of Supplement Use

5.1 While your [wife / daughter (in-law)] has been eating this product(s), has she eaten it every day? If no, why not?
- Has she been eating the full serving every day? If no, why not?
- [only for the choice group] Did it vary by product type?

5.2 How has your [wife / daughter (in-law)] been eating this product? (as a snack, as part of a meal, between meals), has it
a) replaced a whole meal/snack; or
b) replaced some of a meal/snack; or
c) been an addition to the food she normally eats as a meal/snack (extra)?
- [only for the choice group] Did it vary by product type?
- If it has replaced all or some of the food she normally eats, which food, when (i.e. what meal would it replace) and why?

5.3 Have you tasted the product(s)? Has she shared it with you?
- If yes, did you eat it regularly? Why / why not?
- Has anyone else tried the product(s)? If so, who?
- Would you encourage her to share the product(s)? If so, with whom and why?

6 Future Utilization

6.1 What do you think about the fact this product only being provided to pregnant women?
- If this supplement was recommended for your [wife/daughter (in-law)] for the rest of her pregnancy, would you or other members of your household expect to share the product?

- Would your [wife's/daughter (in-law)'s] share of meal food be reduced because she has this product? If yes, by whom?

6.2 If this product continued to be given for free would you want your [wife / daughter (in-law)] to use it for the rest of her pregnancy? Why / why not?

- If you had to pay, would you / your [wife/daughter(in-law)] continue to use the product during pregnancy? Why / why not?

Thank you and close

In-depth interview guide: health professional

1 Pregnancy and Care Seeking

1.1 At what point do you usually find out about women in you community being pregnant Why then?

1.2 When a woman is pregnant, where does she go / who does she go to for information and care? (e.g. traditional healer/FCHV/health centre/ hospital)

1.3 Do you have a role in supporting pregnant women in your community? If yes, what?

- What information do you give them?
- Do you provide or distribute supplements (such as iron / folic acid supplements) to pregnant women?

2 Diet During Pregnancy

2.1 What kinds of food do you think a nutritious diet during pregnancy should include?

2.2 What are the specific foods you believe pregnant women should eat during pregnancy? What are the specific foods you believe pregnant women should not eat during pregnancy?

2.3 Do women try to restrict the amount of food they eat during pregnancy?

- If yes, why? how much do you restrict it?
- From whom/where do women hear about restricting food in pregnancy?

2.4 Who decides what foods and how much a woman should eat during pregnancy? (woman, family members)

2.5 Do women eat snacks during pregnancy?

2.6 As a [health professional] what do you tell pregnant women about diet / nutrition?

- Do women like to receive messages and information about nutrition in pregnancy?
- How do you deliver messages and information to women? What works well?
- Do you speak to anybody else, other than the pregnant woman, to give information? (husband, mother, mother-in-law etc.) What do you tell them?
- Are families and community members receptive to messages and information about nutrition in pregnancy?

3 Availability and access to supplements

3.1 What do you know about micronutrients such as iron or vitamin supplements for pregnant women?
- If yes, what type?

3.2 Are iron or vitamin supplements specifically for pregnant women available in your community?
- If yes, what type?
- When do women take them?
- Where do women get them?
  - If any supplement (tablet) provided by the facility, how many (supplement/tablet) given to a pregnant woman during each visit? Why that amount?

3.3 What do you know about food supplements for pregnant women?
- If yes, what type?

3.4 Are food supplements specifically for pregnant women available in your community?
- If yes, what type?
- When do women take them?
- Where do women get them?

3.5 In your role as a [health professional], do you think women need to take food supplements during pregnancy? why / why not?

3.6 Have you ever recommended or provided food supplements for a pregnant woman? If no, why not? If yes:
- What kind of supplement did you recommend/provide? Why?
- Did anyone ask or tell you to recommend those supplements? Who? What did they tell you?
- What did you tell the woman about the supplement?
- Did they understand how to take the supplement? How do you know?
- What are the biggest barriers preventing the correct use of supplements by pregnant women?

4 <u>Product(s) Opinion from Health Worker Perspective and Distribution</u>

Show the supplement(s) to the health care worker. Indicate to the health worker that these are the types of supplements that we are planning to ask pregnant women to take daily during pregnancy and in the 6 months after delivery.

4.1 What is your opinion of food supplements for pregnant women in general? (positive/negative)
- Would you have any concerns or worries about pregnant women taking a supplement like this? If no, why not? If yes, what would be your concerns?

4.2 In the future, if women were going to use this product during their pregnancy,
- Where would you like them to get it? Why?
- Who would you like her to get it from? Why? (e.g. from FCHV/health centers/ pharmacy/other etc.)
- How often do you think it should be distributed? (e.g. monthly / twice a month / weekly / daily)

4.3 As a health care provider, would you like to have more information about this product? Yes / No. If yes, what and why? If not, why not?
- If all pregnant women were to be given this product, what information should they be given?
- How could pregnant women best be provided with information about the benefits of these supplements and how to take them?

- Is it important for other people in the community to have information about the product? If yes, who and what information should they be given?

4.4 In your role as a [healthcare professional], would you recommend this product to pregnant women in your care? Yes / no, why / why not.
- What can you do to encourage women to use the (food) supplements?
- What can you do to encourage family members support the use (food) supplements in pregnancy?

Thank you and close

Focus group discussion framework: pregnant women

1 Experience of use (during study)

1.1 Having used this product for 8 weeks, what do you think about the product overall?

1.2 Has your opinion of the product changed over the last eight weeks? If yes, how?

1.3 While you have been eating this product, have you eaten it every day? ? If no, why not?
  - While you have been eating this product have you been eating the full serving every day? If no, why not?

1.4 How have you been eating this product? (as a snack, as part of a meal, between meals)
- While you have been eating this product, has it
  a) replaced a whole meal/snack;
  b) replaced some portion of a meal or snack; or
  c) been an addition to the food you normally eat at a meal/snack (extra)?
- If it has replaced all or some of the food you normally eat, which food, when (i.e. what meal did it replace) and why?

1.5 What was the opinion of others in the household about the product?

2 Tasting new flavours (if flavours not available for FGD, skip to Section 3)

Give participants samples of all new product flavours to try and discuss amongst themselves. When all the new flavours have been tasted then ask questions 2.1 – 2.3

2.1 Having tasted the new flavours, which flavour do you prefer? Why?

2.2 Do you have recommendations about the flavours you have tried?

2.3 If there were several flavours of this product available, would you like to receive one flavour or different flavours?
- Would the availability of several flavours of this product make it easier to use (eat daily and eat the full serving) the product for your entire pregnancy?
- How frequently would you want to consume a different flavour? Ex. Every other day, every week, every month etc.

3 Importance of product choice

Explain to participants that there has been one other group in the study who has been eating a different product for these last 8 weeks and give them samples of the products (biscuit and plumpy mum) to try.

Then ask questions 3.1 - 3.3

3.1 If there was more than one product available would you prefer to eat throughout your pregnancy
a) this product (the product that you have been eating for 8-weeks), or
b) the other product ? or
c) would you like to have a choice of the two products?? Why?

3.2 What is your opinion of having a choice of products to eat during pregnancy?

- Would having a choice of products make it easy or difficult to use the product for your entire pregnancy?
- How frequently would you want to change/switch between products? (Every other day/every week/ every month)

3.3 Would you prefer the option to choose the same product that you have been eating in different flavours or would you prefer the option to choose different types of products? Why?

4 Distribution and Information

4.1 If this product were given for free, would you use this product in the future during a pregnancy? Why/why not?

- Would you consume it every day throughout your pregnancy? If not, why not, and how often would you consume it?
- Would you consume it every day for the first six months after you delivered? If not, why not? How often would you consume it?
- Where would you like to get it from? Why? (e.g. from health centers/ pharmacy/other etc.)
- Who would you like to get it from? Why? (e.g. From FCHV/other)
- How often would you like to get the product (e.g. monthly/twice monthly/weekly)?
- If you had to pay would you use this product in the future during a pregnancy? Why / why not?

4.2 If you were to use this product in the future during a pregnancy would you like to have more information about it? Why / why not?

- Where/from whom would you like to hear information about this product?
- If all pregnant women were to be given this product, what information should they be given?
- Is it important for other people in the community to have information about the product? If yes, who and what information should they be given?

4.3 What factors would make it difficult for you to obtain/access the food supplement in the future? What might encourage you or make it easier for you to obtain/access the food supplement in the future?

4.4 What factors would make it difficult for you to consume the (food) supplement (eat daily and eat the full serving) in the future? What might encourage you or make it easier for you to consume the (food) supplement in the future (eat daily and eat the full serving)?

Thank you and close
