# Supplementary material for: Compliance with and acceptability of two fortified balanced energy protein supplements among pregnant women in rural Nepal
Source: Matern Child Nutr. 2021 Dec 15;18(2):e13306. doi: 10.1111/mcn.13306 (PMC8932730; doi:10.1111/mcn.13306)
Supplement: Supplementary file 2 — Supporting information. [file MCN-18-e13306-s001.docx]

**Supporting information file 2: Compliance calculation**

**Measurement of overall compliance over 8 weeks**

1. Packet count method overall compliance (any portion) over 8 weeks among those who were met in person all 8 weeks:

Compliance definition (packet count) = $\frac{\Sigma sachets used}{\Sigma follow up time}$ = $\frac{\Sigma sachets distributed over 8 weeks-\Sigma sachets returned unused over 8 weeks}{last follow up date-first follow up date}$

1. Self-reported overall compliance of FULL portion over 8 weeks among those who were met in person or met over the phone all the 8 weeks:

Compliance FULL portion definition = $\frac{\Sigma number of days reported eating full product}{\Sigma follow up time}$

1. Self-reported overall compliance of ANY portion over 8 weeks among those who were met in person or met over the phone all the 8 weeks:

Compliance ANY portion definition = $\frac{\Sigma number of days reported eating product}{\Sigma follow up time}$
