# Supplementary material for: Compliance with and acceptability of two fortified balanced energy protein supplements among pregnant women in rural Nepal
Source: Matern Child Nutr. 2021 Dec 15;18(2):e13306. doi: 10.1111/mcn.13306 (PMC8932730; doi:10.1111/mcn.13306)
Supplement: Supplementary file 3 — Supporting information. [file MCN-18-e13306-s002.docx]

**Supporting information file 3: Weekly supplement use distribution**

**Table 1a. Distribution of percentage of reports on timing of day when the last supplement was consumed for Lipid-based peanut paste group**

|  | **Week 1** | **Week 2** | **Week 3** | **Week 4** | **Week 5** | **Week 6** | **Week 7** | **Week 8** | **Total** |
| --- | --- | --- | --- | --- | --- | --- | --- | --- | --- |
| **Last ate supplement in the…** |  |  |  |  |  |  |  |  |  |
| Morning | 33.3 | 39.5 | 50.0 | 56.8 | 58.8 | 58.3 | 60.0 | 31.4 | **48.3** |
| Afternoon | 20.5 | 18.4 | 18.4 | 16.2 | 11.8 | 11.1 | 17.1 | 14.3 | **16.1** |
| Evening | 10.3 | 15.8 | 21.1 | 18.9 | 14.7 | 19.4 | 11.4 | 0.0 | **14.0** |
| Night | 33.3 | 26.3 | 7.9 | 8.1 | 11.8 | 8.3 | 5.7 | 5.7 | **13.7** |
| Multiple times | 2.6 | 0.0 | 2.6 | 0.0 | 2.9 | 2.8 | 5.7 | 48.6 | **7.9** |
| **Ate food (meal/snack) as normally do** | 84.6 | 78.9 | 86.8 | 83.8 | 76.5 | 75.0 | 88.6 | 85.7 | **82.5** |

**Table 1b. Distribution of percentage of reports on timing of day when the last supplement was consumed for Biscuit group**

|  | **Week 1** | **Week 2** | **Week 3** | **Week 4** | **Week 5** | **Week 6** | **Week 7** | **Week 8** | **Total** |
| --- | --- | --- | --- | --- | --- | --- | --- | --- | --- |
| **Last ate in the…** |  |  |  |  |  |  |  |  |  |
| Morning | 40.0 | 42.5 | 48.7 | 38.5 | 45.9 | 50.0 | 54.1 | 45.9 | **45.6** |
| Afternoon | 12.5 | 25.0 | 20.5 | 30.8 | 18.9 | 26.3 | 18.9 | 18.9 | **21.5** |
| Evening | 12.5 | 5.0 | 7.7 | 10.3 | 16.2 | 7.9 | 10.8 | 2.7 | **9.1** |
| Night | 7.5 | 2.5 | 0.0 | 2.6 | 2.7 | 2.6 | 5.4 | 5.4 | **3.6** |
| Multiple times | 27.5 | 25.0 | 23.1 | 17.9 | 16.2 | 13.2 | 10.8 | 27.0 | **20.2** |
| **Ate food (meal/snack) as normally do** | 55.0 | 62.5 | 53.8 | 85.7 | 62.2 | 68.4 | 78.4 | 64.9 | **65.1** |
